# Long-term treatment of phenylketonuria with a new medical food containing large neutral amino acids

**DOI:** 10.1038/ejcn.2016.166

**Published:** 2016-09-14

**Authors:** D Concolino, I Mascaro, M T Moricca, G Bonapace, K Matalon, J Trapasso, G Radhakrishnan, C Ferrara, R Matalon, P Strisciuglio

**Affiliations:** 1 Department of Medical and Surgical Science, Pediatrics Unit, University 'Magna Graecia', Catanzaro, Italy; 2 Department of Health and Human Performance, University of Houston, Houston, TX, USA; 3 Department of Pediatrics, University of Texas Medical Branch, Galveston, TX, USA; 4 Department of Public Health and Infectious Diseases, Sapienza University, Rome, Italy; 5 Department of Translational medical Science, Section of Pediatrics, Federico II University, Naples, Italy

## Abstract

**Background/Objectives:**

Phenylketonuria (PKU) is an autosomal recessive disease caused by deficient activity of phenylalanine hydroxylase. A low phenylalanine (Phe) diet is used to treat PKU. The diet is very restrictive, and dietary adherence tends to decrease as patients get older. Methods to improve dietary adherence and blood Phe control are continuously under investigation.

**Subjects/Methods:**

A new formula Phe-neutral amino acid (PheLNAA) has been tested in this study with the purpose of improving the compliance and lowering blood phenylalanine. The formula has been tested for nitrogen balance, and it is nutritionally complete. It is fortified with more nutritional additives that can be deficient in the PKU diet, such as B12, Biotin, DHA, Lutein and increased levels of large neutral amino acids to help lower blood Phe. The new formula has been tested on 12 patients with a loading test of 4 weeks.

**Results:**

Fifty-eight percent of patients had a significant decline in blood Phe concentration from baseline throughout the study. The PheLNAA was well tolerated with excellent compliance and without illnesses during the study.

**Conclusions:**

In conclusion, the new formula is suitable for life-long treatment of PKU, and it offers the PKU clinic a new choice for treatment.

## Introduction

Phenylketonuria (PKU) is caused by deficiency of the enzyme phenylalanine hydroxylase (PAH). This enzyme deficiency results in the inability to convert Phe to tyrosine, leading to an increased concentration of phenylalanine in the blood and central nervous system. The clinical features of untreated PKU include mental retardation, pigment dilution, microcephaly, seizure disorder and eczema.^1^ A diet restricted in phenylalanine (Phe) improved the clinical outcome of patients with PKU in the early trials of Bickel *et al.*^2^ The National Collaborative Study for PKU documented safe blood Phe concentrations for treatment and determined that discontinuing diet treatment around the age of 6 years leads to a decline in intelligence quotient, poor school performance, decline in executive functioning and changes in the white matter of the brain.^3, 4, 5, 6, 7, 8, 9, 10, 11, 12, 13, 14, 15, 16, 17, 18^ This resulted in reassessment of the diet discontinuation policy, and the concept of 'diet for life' emerged.

Treatment for PKU with Phe-free medical food and measured servings of foods with low protein should be continued for life. Items such as low-protein bread, noodles or cookies, sodas and hard candy can be used as fillers. The blood Phe concentration often increases above the desired treatment goal as patients get older because of the limited number and serving sizes of foods available for older children on the diet. Continued efforts are being made to improve treatment for patients with PKU to better regulate blood Phe levels.

A synthetic version of tetrahydrobiopterin (BH4), the cofactor for PAH, can reduce Phe levels in about one-third of patients with PKU.^19^ This can result in improved concentration and executive functioning, but even with BH4 supplementation blood Phe levels can remain above levels targeted, and dietary treatment should be continued.^20^

Large neutral amino acids (LNAA) can be added to the medical food to compete with Phe for the same transporter in the gastrointestinal (GI) tract and blood–brain barrier (BBB).^21^ A new medical food, PheLNAA, was developed with higher concentrations of LNAA fortified with vitamins that are more abundant in high-protein foods. Other nutrients include Lutein, an antioxidant found in high concentration in the retina, and microalgae docosahexaenoic acid (DHA), important for the development of the brain. Increased amounts of biotin, vitamin B12 and folate have also been added. This report investigates the acceptability, tolerance and blood Phe control of the new medical food, PheLNAA.

## Materials and methods

### Recruitment and study population

**Figure 1:**
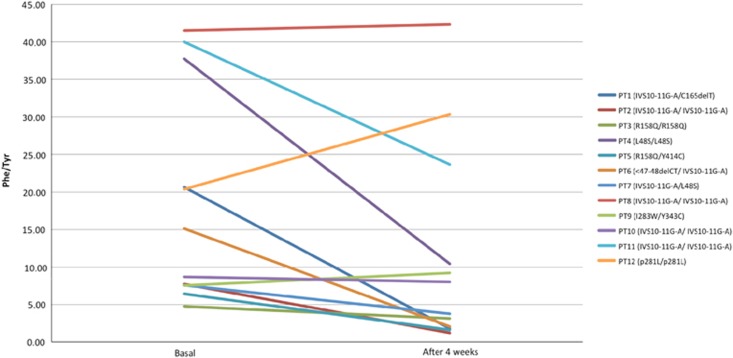
Phe/Tyr ratio at the basal time and after 4 weeks for the 12 patients; in the legend is shown (enclosed in parentheses) the genotype of each patient according to Guzzetta *et al.*^22^

Subjects with PKU in dietary therapy were recruited from the Department of Pediatrics, University of Catanzaro, Italy, and were asked to participate in a research protocol to evaluate the acceptability and tolerance of a new medical food, PheLNAA, and its effectiveness in reducing the concentration of blood Phe. The criteria for inclusion in the study were a diagnosis of mild or classical PKU identified by neonatal screening, dietary therapy from the first month of life to the age of 8 years or older, baseline blood Phe concentration>6 mg/dl (>360 μmol/l) and intelligence quotient in the normal range. All patients in the study were genotyped according to Guzzetta *et al.*^22^ (Figure 1). The exclusion criteria were the presence of other diseases, malabsorption or women who may become pregnant. Eligible patients who agreed to participate, or their parents or legal representative, signed an informed consent form approved by the Institutional Review Board and Ethics Committee at the University of Catanzaro. All procedures were in accordance with the ethical standards of the responsible committee on human experimentation and with the Helsinki Declaration of 1975, as revised in 1983.

### Study design

Baseline values were established 3 weeks before the start of the study. During this time, weekly blood Phe and Tyrosine (Tyr) levels were taken 2 h postprandial, and dietary intake was recorded for 3 days before blood draw. These same measures were continued throughout the loading study period. A dietitian calculated the total dietary intake and recorded the dietary intake of Phe, Tyr, protein and energy; blood Phe and Tyr results were also recorded. Growth data and blood chemistry (electrolytes, BUN, total protein and albumin) were ascertained at the beginning and after 4 weeks of treatment.

A total of 0.5 g/kg/day of the new medical food (Table 1), PheLNAA sachets, produced by MOVISCOM in Rome, Italy, was divided into three doses and administered before main meals for 4 weeks. No other special supplements were given to the patients. Patients who did not initially respond to the 4-week-treatment period continued to receive the study product for an additional 2 weeks to test whether a longer treatment would provide some result.

Each study subject was advised by a dietitian on how much Phe to take during the study. This dietitian then calculated the actual dietary intake of Phe and recorded the blood Phe and Tyr concentrations.

The primary end point was to decrease Phe levels by 30% or more from baseline and then to lower the Phe/Tyr ratio after 4 weeks (responders) or 6 weeks (not responders) of treatment.

Blood Phe and Tyr concentrations were taken and diet diaries accompanied blood specimens. Patients were instructed to contact the clinic for any illness or unusual complaints during the study.

### Statistical analysis

Preliminarily, a descriptive analysis has been performed; in particular, averages, medians, s.d., range and percentage variation of the variables considered in the observation times have been computed.

Subsequently, noting the non-compliance of the data to the normal distribution and also taking into account the small sample size, it was decided to use non-parametric statistical tests. In particular, for the assessment of the variations between the two times, we applied the statistical test based on ranks for paired data of Wilkoxon. As usual, it fixed an error of the first type (α) equal to 0.05, therefore considering as significant those tests presenting a *P*<0.05.

Analyses were performed with 'SPSS Statistics', IBM, Armonk, NY, USA.

## Results

Sixteen patients participated in the trial. Four patients dropped out after the first week because of non-compliance with the study protocol. The remaining 12 patients (three with mild PKU and nine with classic PKU, three males and nine females), with a mean age of 14.8 years, were analyzed (Table 2). The patients were of a mean height of 149.25 cm (±14.21), in a range of 49, and a mean weight of 45.53 kg (±12.17) (whereas the median weight was 47.15 in a range of 40). As regards the body mass index, the mean value was 20.21 (±3.21), in a range of 10.90.

Table 3 shows that the blood Phe concentration decreased, from an average of 15.15, at the basal time (Phe T0), to 11.15 after 4 weeks of treatment (Phe T1), with a mean percentage variation of −31.38%. Moreover, we have tested the difference between Phe T0 and Phe T1, which gave statistically significant results (*P*=0.033).

With regard to Tyrosine concentration, we have a basal mean value (Tyr T0) of 1.14, and a mean value of 1.63 after 4 weeks of treatment (Tyr T1). The mean percentage variation increased by 45.31%, and also in this case the difference between Tyr T0 and Tyr T1 was statistically significant (*P*=0.034).

Finally, we have analyzed the ratio between the level of Phe and Tyr at the basal time and after 4 weeks (Figure 1). We can observe a decrease in this ratio. In fact, the mean percentage variation is of −39.18%. The difference between the two ratio results was statistically significant at the level of 0.05. No correlation was observed between the severity of mutations and response to PheLNAA supplementation.

**Figure 2:**
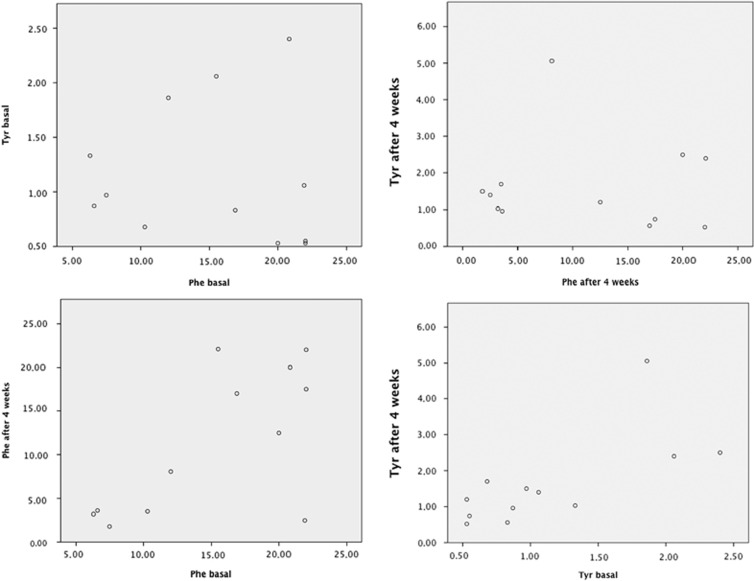
Phe and Tyr levels for each patient.

In Figure 2, we have reported the level of Phe and Tyr at the basal time and after 4 weeks of treatment for each patient.

No patients showed intolerance or illness during the study, and growth remained normal for all children included in the study. The electrolytes, total protein and albumin remained normal before and at the end of the 4 weeks of therapy (data not shown).

## Discussion

Diet for life is the accepted treatment for PKU. The lack of diet adherence in the treatment of PKU is of concern. The NIH National Consensus Committee for Treatment of PKU^23^ in the United States most recently recommended target blood Phe concentrations of <6 mg/dl (360 μmol/l) for all ages. Difficulty in maintaining blood Phe in the recommended range in adolescents has been documented.^4, 6, 7^ Dietary treatment for PKU requires intake of a Phe-free medical food and measured servings of Phe-containing foods to maintain blood Phe concentrations in the desired treatment range. The amount and types of foods that can be eaten are limited, and this makes it difficult for adolescents to comply with diet. Methods to improve dietary adherence in PKU patients and improve blood Phe control are greatly needed.

This study utilized the newly formulated medical food, PheLNAA sachets. It contains increased concentrations of large neutral amino acids compared with other medical foods used for the treatment of PKU. In addition, it contains more vitamins and other nutritional additives that can be deficient in the PKU diet, such as vitamin B12, Biotin, DHA and Lutein. The LNAA can help reach target blood Phe levels by blocking some of the transport of Phe in the GI tract and across the BBB. In the GI tract, LNAA is transported by a carrier protein with *K*_m_ that is two orders of magnitude higher than that of the BBB carrier protein.^24^ According to the experiments of Hidalgo *et al.,*^24^ a significant inhibition of Phe transport requires 100-fold (1 mm) LNAA, as dictated by the *K*_m_ equation for affinity of LNAA to the GI carrier protein. For example, at such concentrations, leucine inhibits Phe transport by 55%, tyrosine by 45% and the cationic amino acid lysine by 50%.^24^ Under physiological conditions, competition of LNAA with Phe is not likely to occur in the GI tract. However, by increasing the concentration of LNAA and lysine, while the concentration of Phe is unchanged or reduced, competition with the GI transporter can be achieved. The transport of LNAA to the brain is mediated by a carrier protein with the lowest *K*_m_ for Phe. The *K*_m_ equation predicts that if the plasma level of one of the large neutral amino acids has a lower *K*_m_, then that amino acid will not compete effectively for the carrier protein.^25, 26, 27, 28^ There are reports on mice and some patients with PKU who have lower concentrations of tyrosine, tryptophan and branched chain amino acids^29^ in the brain as a result of the elevated blood Phe crossing the BBB. This may be due to little competition from the other large neutral amino acids. Mice with PKU were shown to have leucine concentration that is lower in the brain because of the preference for Phe for transport across the BBB. When fed mouse chow with 16.7% LNAA, these mice had a statistically significant decrease in blood Phe concentration.^30, 31^ This suggests that a competition with the transport of Phe can be attained with high levels of LNAA in the GI tract, satisfying the concentrations required by Km for the GI transporter.

In human patients with PKU, we have shown in a double-blind study that blood Phe concentration declines significantly when treated with LNAA.^32^ These studies suggest that adding increased dietary concentration of LNAA for patients with PKU will lead to a reduction in blood Phe concentrations.^32^

The current study utilized PheLNAA sachets, a new medical food that contains increased concentrations of LNAA, and includes some other nutrients needed for treatment of PKU, making it a more complete food (Table 1). There were 16 subjects with PKU recruited for the study; only 12 completed the loading test, as seen in Figure 1. Seven patients successfully improved blood Phe control equal to or greater than the primary end goal set (30% reduction) after 4 weeks of taking PheLNAA sachets. These seven patients showed between 32 and 90% decline in blood Phe concentration from baseline throughout the study. Therefore, it is evident that the increased concentration of LNAA in the PheLNAA medical food greatly inhibited the transport of Phe in the GI tract and to the BBB in these patients. Of the other five patients in our study, two achieved a decrease in blood Phe levels, but it was less than the 30% primary end goal set. The other three patients had an increase in blood Phe levels. Unfortunately, the patients who responded less to the treatment were the older aged children in our study group (data not shown). We speculate that the older aged children can manipulate their intake and follow the diet incoherently, whereas the younger children can follow their diet more strictly with their parents' help.

Overall, the PheLNAA medical food was ranked by the participants in this study, using their previous extensive experience, as having a better taste compared with their past formulas. Ideally, the PheLNAA medical food should be started at a younger age to assuage the non-compliance problem. If children become more familiar with the taste of their medical food at a younger age, it may be easier for them to accept it and adhere to their diet in the long run. When transitioning to the PheLNAA medical food from their old formula it is possible, if needed, to mix the two formulas for the first few days. This is only if the child is used to and demands the old formula; otherwise, it is advisable to completely switch to the new formula immediately. Of course, a dietitian should supervise this transition period to ensure that patients' daily nutritional needs are met. This would eliminate the problem of non-compliance that was experienced by some patients with this study. The patients with PKU would benefit from changing to PheLNAA in childhood, which should lead to improved blood Phe control.

## Figures and Tables

**Table 1:** Composition of PheLNAA for 100 g powder

			Vitamins		
Energy	kcal	293.50	A	mcg	1300
	kJ	1241.26	D_3_	mcg	15.5
			E	mg	20
			K1	mcg	113
			C	mg	100
			B_1_	mg	1.5
Protein equivalent	g	48.94	B_2_	mg	1.8
Carbohydrates	g	15.74	B_6_	mg	5.4
Sugars	g	12.46	B _12_	mcg	9
Fats	g	2.58	Biotin	mcg	150
DHA	mg	610	Choline	mg	650
Amino Acids	g		Inositol	mg	125
Arginine	g	61.73	Niacin	mg	20
Aspartic Acid	g	2.79	Folic acid	mcg	750
Glutamine	g	2.36	Pantothenic acid	mg	8.8
			Minerals		
Isoleucine	g	3.52	Calcium	mg	1600
Leucine	g	3.33	Chromium	mcg	40
Lysine	g	9.79	Copper	mg	1.5
Methionine	g	5.70	Iodine	mcg	200
Proline	g	0.97	Iron	mg	20
Threonine	g	3.45	Magnesium	mg	500
Tryptophan	g	3.15	Manganese	mg	3
Tyrosine	g	3.33	Molybdenum	mcg	90
Valine	g	18.79	Phosphorous	mg	1250
Hystidine	g	1.21	Selenium	mcg	75
			Sodium	mg	40
			Zinc	mg	15
			Lutein	mg	12

Abbreviation: PheLNAA, phenylalanine-neutral amino acid.

**Table 2:** Loading test results and data of the responding patients after 6 months of treatment

*Patients*	*Genotype*	*Basal values*			*After 4 weeks*		*After 6 weeks*	*After 6 months*		
		*Phe blood mg/dl (μmol/l)*	*Tyr blood mg/dl (μmol/l)*	*Phetolerance mg/die*	*Phe blood mg/dl (μmol/l)*	*Tyr blood mg/dl (μmol/l))*	*Phe blood mg/dl (μmol/l)*	*Phe blood mg/dl (μmol/l)*	*Tyr blood mg/dl (μmol/l)*	*Phetolerance mg/die*
1	IVS10-11G-A/C165delT	21.90 (1326)	1.06 (58.5)	285	2.50 (151)	1.4 (77.3)		2.00 (121)	1.40 (77.3)	300
2	IVS10-11G-A/IVS10-11G-A	7.50 (454)	0.97 (53.5)	232	1.80 (109)	1.5 (82.8)		1.50 (91)	0.9 (49.7)	264
3	R158Q/R158Q	6.30 (381.4)	1.33 (73.4)	373	3.20 (194)	1.03 (56.8)		7.40 (448.)	0.79 (43.6)	373^a^
4	L48S/L48S	20.00 (1211)	0.53 (29.3)	338	12.5 (757)	1.2 (66.2)		10.2 (618)	1.2 (66.2)	383
5	R158Q/Y414C	12.00 (727)	1.86 (103)	253	8.10 (490)	5.06 (279.3)		6.20 (375)	0.67 (37.0)	253
6	<47-48delCT/IVS10-11G-A	10.30 (624)	0.68 (37.5)	278	3.50 (212)	1.7 (93.8)		14.7 (890)	1.30 (71.7)	278^a^
7	IVS10-11G-A/L48S	6.60 (400)	0.87 (48.0)	518	3.60 (218)	0.9 (49.7)		3.20 (194)	1.30 (71.7)	550
8	IVS10-11G-A/IVS10-11G-A	22.00	0.53	500	22.00	0.52	19.20			
9	I283W/Y343C	15.50 (938.4)	2.06 (114)	500	22.08 (1337)	2.4 (133)	18.60 (1126)			
10	IVS10-11G-A/IVS10-11G-A	20.80 (1259)	2.4 (133)	391	20.00 (1211)	2.5 (138)	15.80 (956)			
11	IVS10-11G-A/IVS10-11G-A	22.00 (1332)	0.55 (30.4)	296	17.50 (1060)	0.74 (40.8)	19.00 (1150)			
12	p281L/p281L	16.90 (1023)	0.8 (44.2)	458	17.00 (1029)	0.56 (30.9)	23.08 (1397)			

a not adhering to dietary therapy in the last month.

**Table 3:** Descriptive analysis of the blood Phe and Tyr levels

	*Mean*	*s.d.*	*Median*	*Range*	*P*
Phe T0	15.15	6.34	16.20	15.70	0.033
Phe T1	11.15	8.21	10.30	20.28	
% var Phe T1/T0	−31.38	37.36	−35.00	131.04	
Tyr T0	1.14	0.64	0.92	1.87	0.034
Tyr T1	1.63	1.26	1.30	4.54	
% var Tyr T1/T0	45.31	67.83	24.29	204.57	
Phe T0/Tyr T0	18.18	14.04	11.90	36.77	0.050
Phe T1/Tyr T1	11.45	13.45	5.87	41.11	
% var Phe/Tyr T1/T0	−39.18	46.52	−45.72	140.45	
