# Erratum: Long-term treatment of phenylketonuria with a new medical food containing large neutral amino acids

**DOI:** 10.1038/ejcn.2017.98

**Published:** 2017-08-02

**Authors:** D Concolino, I Mascaro, M T Moricca, G Bonapace, K Matalon, J Trapasso, G Radhakrishnan, C Ferrara, R Matalon, P Strisciuglio

**Correction to:** *European Journal of Clinical Nutrition* (2017); **71** (1); 51-55; doi: 10.1038/ejcn.2016.166; published online 14th September 2016

Since the publication of the article, the authors have noticed an error in Table 1. The corrected table can be found below.

**Table 1:** Composition of PheLNAA for 100 g powder

**Energy**	kcal	293.50	**Vitamins**		
	kJ	1241.26	A	mcg	1300
			D_3_	mcg	15.5
Protein equivalent	g	48.94	E	mg	20
Carbohydrates	g	15.74	K_1_	mcg	113
Sugars	g	12.46	C	mg	100
Fats	g	2.58	B_1_	mg	1.5
DHA	mg	610	B_2_	mg	1.8
			B_6_	mg	5.4
			B_12_	mcg	9
**Amino Acids**	g	61.73	Biotin	mcg	150
Arginine	g	2.79	Choline	mg	650
Aspartic Acid	g	2.36	Inositol	mg	125
Glutamine	g	3.52	Niacin	mg	20
Isoleucine	g	3.33	Folic acid	mcg	750
Leucine	g	9.79	Pantothenic acid	mg	8.8
Lysine	g	5.70	**Minerals**		
Methionine	g	0.97	Calcium	mg	1600
Proline	g	3.45	Chromium	mcg	40
Threonine	g	3.15	Copper	mg	1.5
Tryptophan	g	3.33	Iodine	mcg	200
Tyrosine	g	18.79	Iron	mg	20
Valine	g	3.33	Magnesium	mg	500
Hystidine	g	1.21	Manganese	mg	3
			Molybdenum	mcg	90
			Phosphorous	mg	1250
			Selenium	mcg	75
			Sodium	mg	40
			Zinc	mg	15
			**Lutein**	mg	12

Abbreviation: PheLNAA, phenylalanine-neutral amino acid.

The authors apologise for any inconvenience caused by this error.

